# Meta-analysis of defunctioning stoma in low anterior resection with total mesorectal excision for rectal cancer: evidence based on thirteen studies

**DOI:** 10.1186/s12957-014-0417-1

**Published:** 2015-01-24

**Authors:** Wen-long Gu, Sheng-wen Wu

**Affiliations:** Department of Medical Oncology, The Affiliated Jianhu Hospital of Nantong University, Jianhu People’s Hospital, Jianhu, 224700 Jiangsu Province China; Department of General Surgery, The Affiliated Jianhu Hospital of Nantong University, Jianhu People’s Hospital, Jianhu, 224700 Jiangsu Province China

**Keywords:** Defunctioning stoma, Low anterior resection, Meta-analysis, Rectal cancer

## Abstract

**Background:**

Recent studies have shown that a protective stoma can reduce morbidity in low anterior resection for rectal cancer; however, the necessity of it is still controversially discussed.

**Methods:**

We performed this meta-analysis to provide a comprehensive evaluation of the role of defunctioning stoma in low anterior resection for rectal cancer on the rates of anastomotic leakage and reoperation related to leakage with or without defunctioning stoma by calculating the pooled risk ratio.

**Results:**

Studies and relevant literature published between 2004 and 2014 regarding the construction of a protective stoma after low anterior resection were searched though PubMed and EMBASE databases. Finally, a total of 13 studies including 8,002 patients were included in this meta-analysis. The results indicated that protective stomas significantly reduced the rate of postoperative anastomotic leakage and reoperation after low anterior rectal resection. The pooled risk ratios were 0.47 (95% CI: 0.33–0.68, *P* <0.0001) and 0.36 (95% CI: 028–0.46, *P* <0.00001), respectively.

**Conclusions:**

The findings from this present meta-analysis suggest that a defunctioning stoma could effectively reduce the clinical consequences of anastomotic leakage and reoperation, it is recommended in patients undergoing low rectal anterior resection for rectal cancer.

## Background

With better equipment and improved surgical techniques, low anterior resection with a low colorectal or coloanal anastomosis has become the technique of choice for rectal cancer, allowing a safe performance of anastomosis at a lower level in a way that the anal sphincter is saved [1]. Additionally, widespread propagation of standardized total mesorectal excision has improved overall survival [2]. However, total mesorectal excision may be associated with an increased risk of developing anastomotic leakage with attendant morbidity and mortality in the postoperative period [3]. Leaks may be associated with decreased local control and survival [4,5] and it is still one of the most fatal complications that occur after low anterior resection [6]. Even experienced surgeons sometimes find it difficult to predict which patient will have an anastomotic leak, and know that leaks may occur even when the anastomosis is technically sound and the risk factors for leakage are absent. When leakage ensues, it may increase morbidity, mortality, prolong the duration of hospital stay, and affect the short- or long-term quality of life [7,8]. Therefore, the rate of anastomotic leakage has been considered as one of the quality indicators of surgical performance.

Several retrospective or non-randomized prospective studies have shown that the absence of a protective stoma is a risk factor for leakage after low anterior resection [9], but others have disputed this [10]. Some surgeons use a protective stoma after low anterior resection to prevent the occurrence of anastomotic leak because it is believed that by diverting the fecal stream and keeping the anastomosis free of material, leakage will be less likely. While other surgeons reported that covering a protective stoma had no influence on anastomotic leak and reoperation rate, and the complications that can be caused by the stoma itself should not be ignored [11-17]. Although a defunctioning stoma is widely performed in low anterior resection for rectal cancer, it is still not clear whether protective stoma is useful for patients. Therefore, the role of defunctioning stoma in rectal cancer surgery is controversial. The primary aim of this meta-analysis was to evaluate the validity for low anterior resection with and without the creation of a defunctioning stoma.

## Methods

### Search strategy

Two bibliographic databases (PubMed and Embase) were searched for all relevant literature, including articles referenced in the publications. The medical subject headings (MeSH) and keywords searched for individually and in combination were as follows: “stoma”, “defunctioning stoma”, “protective stoma”, “low anterior resection”, “rectal cancer”, and “anastomotic leakage”. The search ended in January 31^st^ 2014, and no lower date limit was used. Bibliographies cited in an identified article were also searched manually to retrieve other suitable studies. We also screened the references of the relevant studies to check for potentially relevant articles.

### Inclusion and exclusion criteria

Criteria for eligibility of a study included in this meta-analysis were i) studies that compared low anterior resection with or without a protective stoma; ii) recent clinical trials from 2004 to 2014. When a study reporting the same patient cohort was included in several publications, only the most recent or complete study was selected. Exclusion criteria included i) case reports, letters, and reviews without original data; ii) non-English papers; iii) animal or laboratory studies; or iv) articles that were not full-text and non-comparative studies. To avoid the influence of redundant studies, we checked all of the authors and organizations, and evaluated the accrual period and community of patients enrolled for each study.

### Data extraction

Extracted data were crosschecked between the two authors to rule out any discrepancy. The following data was independently extracted for each included study: first author’s surname, publication year, sample size, and the number of patients that developed an anastomotic leak and needed a reoperation related to leakage after low anterior resection with or without protective stoma. Disagreements were discussed by the authors and resolved by consensus.

### Statistical analysis

Statistical analysis was carried out using the Review Manager 5.2. A pooled risk ratio (RR) with 95% confidence intervals (CIs) was used to assess outcomes of the studies. *I*^2^ statistics was used to evaluate the between-study heterogeneity analysis in this meta-analysis [18]. The random effects model was used when an obvious heterogeneity was observed among the included studies (*I*^2^ > 50%). The fixed effects model was used when there was no significant heterogeneity between the included studies (*I*^2^ ≤ 50%). Publication bias was estimated using a funnel plot with an Egger’s linear regression test; funnel plot asymmetry on the natural logarithm scale of the RR was measured by a linear regression approach.

### Ethical standards

This study complies with current laws of china.

## Results

### Eligible studies

In total, 13 studies were included in the meta-analysis [19-31], all of which were published between 2004 and 2014. There were four randomized controlled trials [20,26,27,31] and nine non-randomized studies with a total population of 8,002 patients, of whom 3,562 had a protective stoma and 4,440 did not. The sample size of the trials ranged from 34 to 1,958. All studies reported the number of patients who developed an anastomotic leak and required a reoperation after low anterior resection or ultralow anterior resection. Table 1 lists the main characteristics of the 13 studies included in this analysis.

**Table 1:** **Main characteristics of the 13 included studies**

			**N**			**Leakage**		**Reoperation**	
**Author**	**Year**	**No. of patients**	**Stoma**	**No stoma**	**Type of operation**	**Stoma**	**No stoma**	**Stoma**	**No stoma**
Beirens et al. [19]	2012	1,912	1,183	729	LAR	51	74	40	69
Chude et al. [20]	2008	256	136	120	LAR	3	12	0	2
Eriksen et al. [21]	2005	1,958	622	1,336	LAR	64	164	NA	NA
Gong et al. [22]	2012	62	36	26	uLAR	0	5	0	2
Karahasanogl et al. [23]	2011	77	23	54	LAR	0	3	NA	NA
Lefebure et al. [24]	2008	132	42	90	LAR	3	10	1	5
Ma et al. [25]	2013	56	30	26	LAR	2	7	0	5
Matthiessen et al. [26]	2004	432	72	360	LAR	11	42	1	32
Matthiessenet al. [27]	2007	234	116	118	LAR	12	33	12	32
Nurkin et al. [28]	2013	1,791	958	833	LAR	17	26	37	63
Seo et al. [29]	2013	836	246	590	uLAR	1	22	NA	NA
Shiomi et al. [30]	2010	222	80	142	LAR	3	17	0	14
Ulrich et al. [31]	2009	34	18	16	LAR	1	6	0	6

LAR, Low anterior resection; uLAR, Ultralow anterior resection; NA, Not applicable.

### Meta-analysis

**Figure 1:**
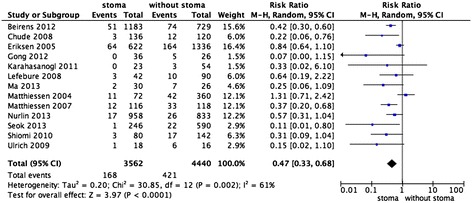
**Forest plot for a comparison of the study outcomes of low anterior resection with or without stoma vs. anastomotic leakage.** Risk ratios are shown with 95% CIs.

**Figure 2:**
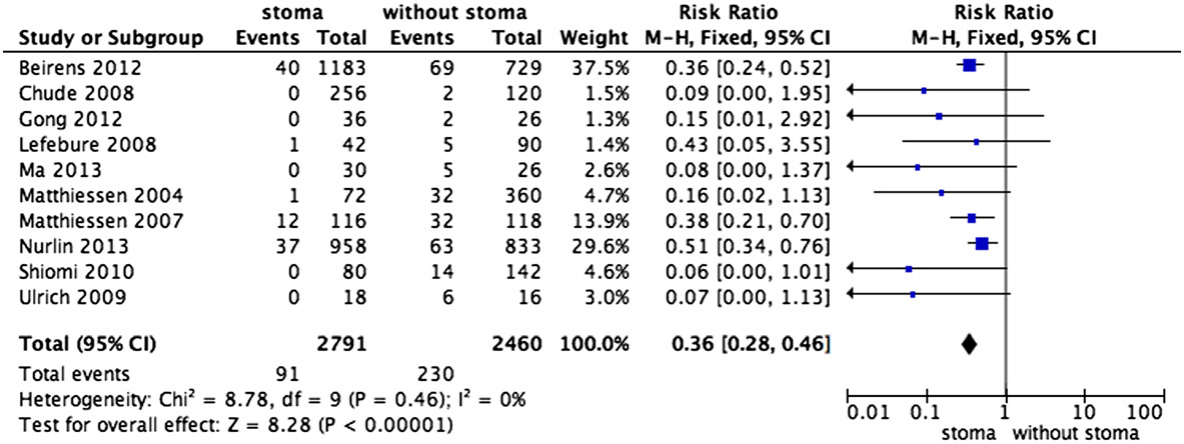
**Forest plot of the study outcomes of low anterior resection with or without stoma vs. reoperation rate.** Risk ratios are shown with 95% CIs.

There was obvious between-study heterogeneity among the 13 included studies (I^2^ = 61%), thus the random effects model was used to calculate the pooled RRs with corresponding 95% CIs. The present meta-analysis demonstrated that the absence of a protective stoma was associated with a higher incidence of anastomotic leak and reoperation, with pooled RRs of 0.47 (95% CI: 0.33–0.68, *P* <0.0001, Figure 1) and 0.36 (95% CI: 028–0.46, *P* <0.00001, Figure 2), respectively. This revealed that a statistically significant advantage was conferred by a protective stoma in patients undergoing low anterior resection.

### Publication bias

**Figure 3:**
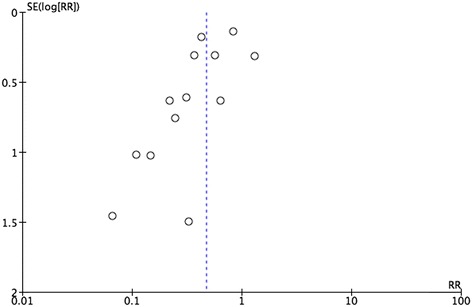
**Funnel plot for the publication bias.**

Funnel plot and Egger’s test were used to evaluate the publication bias of the included studies. The shape of the funnel plot for the meta-analysis of studies on postoperative anastomotic leakage demonstrated obvious asymmetry (Figure 3).

## Discussion

With the development of rectal cancer and the improvement of medical instruments, together with higher requirements by patients for the quality of post-surgical life, ultralow anterior rectal resection has become the major low sphincter preserving procedure. However, this procedure can also increase the risk of anastomotic leakage [9].

The occurrence of adverse intraoperative events was identified as an important risk factor, as previously been indicated by Matthiessen et al. [26]. Moreover, a long operation time and major perioperative bleeding are inter-correlated factors previously discussed in other studies as both single [32] and combined [33] risk factors. Preoperative radiotherapy appeared to be a predisposing factor for leakage in earlier studies of consecutive cases [34]. In addition to the already known risk factors, such as male gender and low anastomoses, other risk factors, such as type of anastomosis and intra-abdominal drainage, have also been identified. Jestin et al. [35] observed that adverse intraoperative events, a long duration of surgery, and major bleeding, all of which are indicators of complicated surgery, increase the risk of leakage. When these occur, the events have been associated with both reduced disease-free survival and overall survival rates.

Defunctioning stoma in low anterior resection has been considered to decrease the leakage rate and its fatal consequences. However, the value of a protective stoma has been the subject of controversy for many years. In previous publications, overall leakage and reoperation rates have been shown to be similar in patients with or without a protective stoma [36]. In addition, ostomy construction and closure is associated with considerable morbidity and increased costs [37]. Potential disadvantages of a protective stoma include the need for re-operation, longer hospital stay, and ostomy-related complications, such as dehydration, which could prove fatal. Therefore, the benefits of a protective stoma in decreasing the rate of anastomotic leakage have to be balanced against the morbidity of its construction and closure [38]. Nevertheless, the benefits conferred by a protective stoma have not been unequivocally demonstrated. To further evaluate this argument, we performed the present meta-analysis. The straightforward conclusion from the 13 included studies was that a protective stoma after a low anterior resection significantly reduces the rate of anastomotic leakage and the number of reoperations related to leakage.

However, we still should regard these outcomes with caution and evaluate them critically for the following reasons. Firstly, funnel plots were performed to evaluate publication bias. The shape of the funnel plot for the meta-analysis of studies on postoperative anastomotic leakage demonstrated obvious asymmetry in their results. We interpreted this asymmetry as different case selection, such as elective or emergency low anterior resection for rectal cancer. Due to the limitations in terms of medical ethics, not all of the trials were randomized controlled trials and the sample size in some studies was rather low, rendering the overall methodological quality and reporting of the included studies rather poor. Secondly, considerable selection bias existed in some of the included studies. Surgeons relied on their personal experience to predict the patients who were at high risk of an anastomotic leakage, which may have been inaccurate, leading to a selection bias of those who underwent stoma formation. Thirdly, the original purpose of the defunctioning stoma was to minimize the rate of anastomotic leakage, but morbidity and mortality can occur at the time of stoma closure. Furthermore, patients who received a protective stoma require readmission for the stoma closure [16].

## Conclusions

In conclusion, despite the inherent limitations of meta-analysis on stoma literature, this meta-analysis, representing a quantified synthesis of all published studies of protective stoma, has shown that a defunctioning stoma significantly reduces the rate of anastomotic leakage and reoperation in patients that receive low anterior resection for rectal cancer. Morbidity associated with protective stoma and complications of stoma closure are negligible compared to the reoperations required for anastomotic leakage in the absence of protective stoma. Therefore, a defunctioning stoma can be useful for patients undergoing rectal surgery, and is recommended during a low anterior resection for rectal cancer.

### Consent

Written informed consent was obtained from the patients for the publication of this report and any accompanying images.

## Abbreviations

CIs: Confidence intervals
RR: Risk ratio
